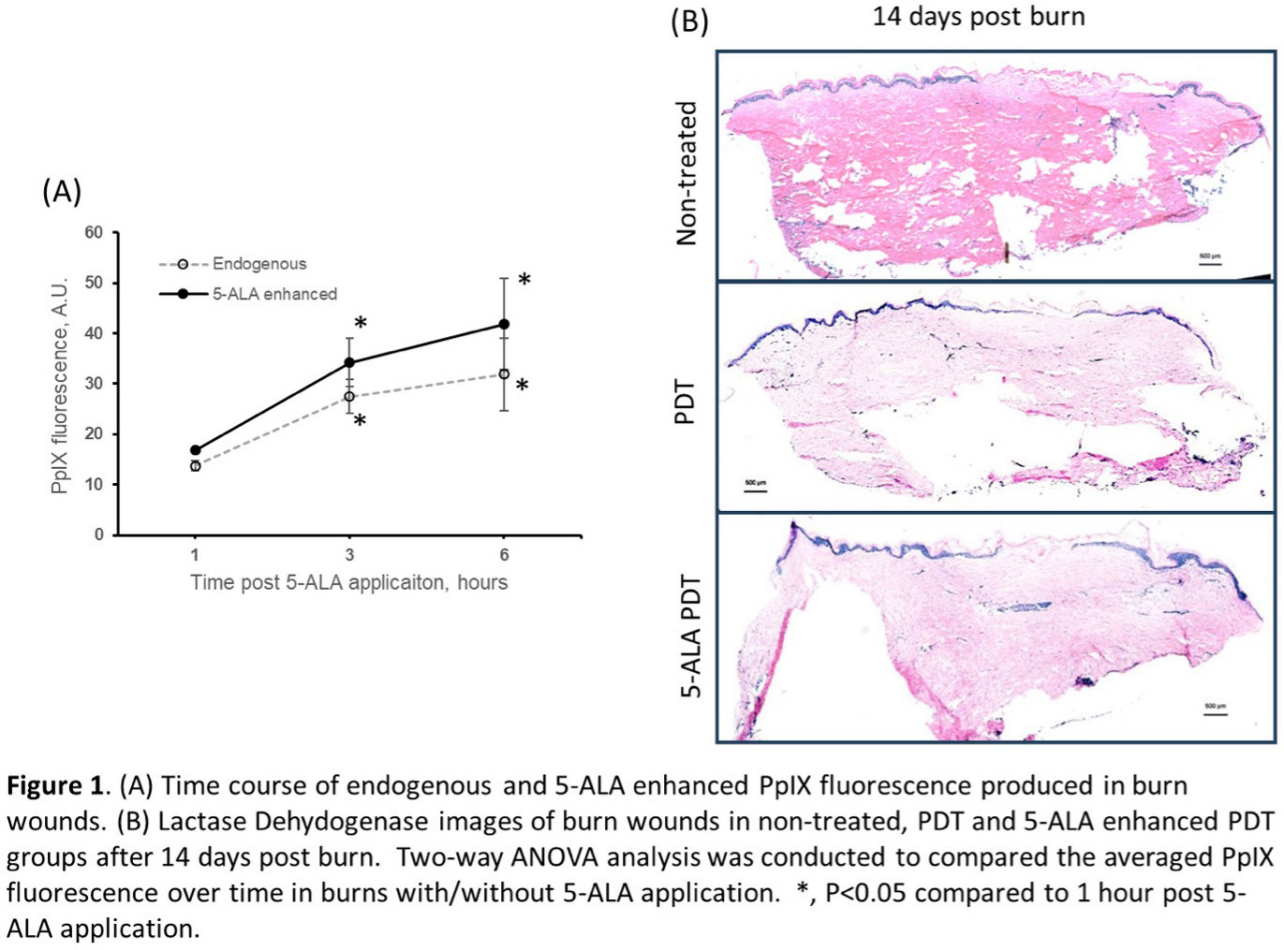# 551 Protoporphyrin IX Based- photodynamic Therapy Enhances Burn Wound Healing in Ex Vivo Human Skin

**DOI:** 10.1093/jbcr/iraf019.180

**Published:** 2025-04-01

**Authors:** Aiping Liu, Marien Ochoa, Emily Klossowski, Bailey Donahue, Joana Pashaj, Mary Junak, Brian Pogue, Angela Gibson

**Affiliations:** University of Wisconsin - Madison; University of Wisconsin - Madison; University of Wisconsin - Madison; University of Wisconsin - Madison; University of Wisconsin - Madison; University of Wisconsin - Madison; University of Wisconsin - Madison; University of Wisconsin School of Medicine and Public Health

## Abstract

**Introduction:**

Photodynamic therapy (PDT) is widely used to treat skin tumors and infections where protoporphyrin IX (PpIX) is a commonly used as the photosensitizer. PpIX is synthesized through the heme pathway from its precursor 5-aminolevulinic acid (5-ALA) endogenously and can be induced through topical application of 5-ALA. In contrast to traditional PpIX-PDT, low-dose PpIX-PDT utilizes lower concentrations of photosensitizer and/or irradiation doses to generate a lower level of ROS, which has been shown to promote burn wound healing in rodents. However, translation of this therapy in human burns remains unknown. This pilot study aimed to evaluate PpIX-PDT in human burn wound healing ex vivo.

**Methods:**

Partial thickness burns were created on ex vivo human skin using a customized burn device. Three 5-ALA application groups and three groups without 5-ALA were used to study the temporal production of PpIX. The 5-ALA groups had PpIX imaged at 1, 3 or 6 hours after topical application of 20% 5-ALA solution along with the corresponding groups without 5-ALA application. To study the effect of PpIX-PDT on burn wound healing, PDT with or without application of 5-ALA, and a non-treated group were evaluated. 3 hours after 20% ALA application (or no treatment controls), the PDT groups were illuminated by red light (630 nm) using a light-emitting diode lamp on the burn region at an energy density of 20 J/cm2. Tissue biopsies were then cultured for 14 days, processed for histology and stained for Lactase Dehydrogenase to evaluate cell viability and healing.

**Results:**

PpIX fluorescence was significantly higher after 3 hours of 5-ALA application than that of 1 hours of application. PpIX fluorescence at 6 hours of 5-ALA application was not significantly different from that of 3 hours of application (Figure 1A). Interestingly, without 5-ALA application, PpIX was produced endogenously in burn wounds and increased with time. 20% 5-ALA application non-significantly enhanced PpIX production in burn wounds. After 14 days, both PDT groups demonstrated enhanced reepithelization in burn wounds without overt cytotoxicity compared to the non-treated control (Figure 1B). Healing after PpIX PDT treatment does not require exogenous 5-ALA application.

**Conclusions:**

This pilot study demonstrated efficacy of PpIX PDT in burn wound healing in human skin. In addition, our data showed that endogenously PpIX PDT has similar effects as 5-ALA enhanced PDT in burn wound healing. We will further optimize PDT parameters such as increasing 5-ALA concentration, light dose and treatment scheme using this ex vivo human skin model of burn.

**Applicability of Research to Practice:**

PpIX-PDT is a low-cost medical technology that has the potential to accelerate healing, leading to improved long-term wound outcomes and quality of life.

**Funding for the Study:**

N/A